# Loss-Based Attention for Interpreting Image-Level Prediction of Convolutional Neural Networks

**DOI:** 10.1109/TIP.2020.3046875

**Published:** 2021-01-11

**Authors:** Xiaoshuang Shi, Fuyong Xing, Kaidi Xu, Pingjun Chen, Yun Liang, Zhiyong Lu, Zhenhua Guo

**Affiliations:** J. Crayton Pruitt Family Department of Biomedical Engineering, University of Florida, Gainesville, FL 32611 USA, and also with the National Center for Biotechnology Information (NCBI), National Library of Medicine (NLM), National Institutes of Health (NIH), Bethesda, MD 20892 USA; Department of Biostatistics and Informatics, Colorado School of Public Health, University of Colorado Denver, Denver, CO 80204 USA; Department of Electrical Computer Engineering, Northeastern University, Boston, MA 02115 USA; J. Crayton Pruitt Family Department of Biomedical Engineering, University of Florida, Gainesville, FL 32611 USA; J. Crayton Pruitt Family Department of Biomedical Engineering, University of Florida, Gainesville, FL 32611 USA; National Center for Biotechnology Information (NCBI), National Library of Medicine (NLM), National Institutes of Health (NIH), Bethesda, MD 20892 USA; Shenzhen International Graduate School, Tsinghua University, Shenzhen 518055, China

**Keywords:** Deep neural networks, loss-based attention, patch mining, weighted sum

## Abstract

Although deep neural networks have achieved great success on numerous large-scale tasks, poor interpretability is still a notorious obstacle for practical applications. In this paper, we propose a novel and general attention mechanism, loss-based attention, upon which we modify deep neural networks to mine significant image patches for explaining which parts determine the image decision-making. This is inspired by the fact that some patches contain significant objects or their parts for image-level decision. Unlike previous attention mechanisms that adopt different layers and parameters to learn weights and image prediction, the proposed loss-based attention mechanism mines significant patches by utilizing the same parameters to learn patch weights and logits (class vectors), and image prediction simultaneously, so as to connect the attention mechanism with the loss function for boosting the patch precision and recall. Additionally, different from previous popular networks that utilize max-pooling or stride operations in convolutional layers without considering the spatial relationship of features, the modified deep architectures first remove them to preserve the spatial relationship of image patches and greatly reduce their dependencies, and then add two convolutional or capsule layers to extract their features. With the learned patch weights, the image-level decision of the modified deep architectures is the weighted sum on patches. Extensive experiments on large-scale benchmark databases demonstrate that the proposed architectures can obtain better or competitive performance to state-of-the-art baseline networks with better interpretability.

## I. Introduction

OVER the past few years, convolutional neural networks (CNNs) have exhibited powerful capability on discriminative feature extraction and achieved tremendous success on many computer vision and pattern recognition tasks [1]–[5]. However, CNNs still confront several limitations. One notorious drawback is poor interpretability, e.g. it is difficult to understand how they reach their decisions, and which objects or their parts determine the image-level prediction [6], [7].

To enhance the interpretability of CNNs, most existing studies focus on understanding the representations of pre-trained CNNs or learning CNNs with interpretable/disentangled middle- or high-layer representations [8]. These methods usually collect the evidence from feature maps or filters to discover the significant image regions or object parts for an image-level decision, instead of directly and explicitly explaining the significant parts during training. Additionally, they are often based on current popular CNNs, most of which do not maintain the spatial relationship of features in one image because of pooling. This would make the effect of any image part on a hidden activation highly depend on other parts, thereby increasing the difficulty of interpretation, e.g. which parts determine the image-level prediction. To better understand or preserve the spatial relationship of features, capsule networks [9], [10], which utilize vector-output capsules to replace the scale-output feature detectors of CNNs, employ dynamic routing to substitute one popular operator, max-pooling. Because max-pooling only extracts the most meaningful information in a local pool and potentially loses some useful information. Nevertheless, dynamic routing is an extremely expensive procedure, with consuming very high computation and memory costs, especially for multiple routing layers spending much training and inference time [11]. Additionally, dynamic routing cannot explicitly take into account the significance of patches in an image, because it directly calculates the class probability of each capsule instead of patches. However, discovering significant patches in one image is beneficial to the understanding of the image-level decision and even the improvement of image prediction accuracy, because some patches might contain the significant objects or their parts.

Attention mechanisms [12] can be utilized to discover the significant patches, because they are capable to assign large weights to significant patches and meanwhile provide small weights to trivial patches. However, current attention mechanisms [13] are widely applied to nowadays popular CNNs, such as VGG [14], GoogleNet [15] and ResNet [5], which often do not preserve the spatial relationship of patches in an image. More importantly, they usually learn patch weights and image prediction with different layers and parameters, so that the image classification accuracy significantly depends on the effectiveness of learned patch weights. Unfortunately, attention mechanisms easily assign large weights to trivial patches, thereby potentially decreasing model performance.

**Fig. 1.**
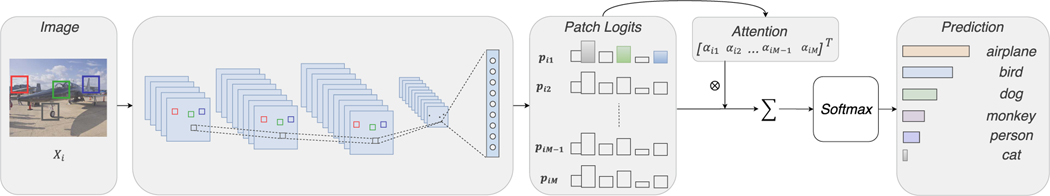
The idea of the proposed convolutional architecture using a weighted sum of patches for the image-level decision. We remove max-pooling or stride operations in convolutional layers to preserve the spatial relationship of patches, and we only utilize stride in one convolutional layer to extract patch features for patch logit generation. A detailed convolutional architecture is displayed in the middle panel of Fig. 3. ${\left[\alpha_{i1},\cdots ,\alpha_{iM}\right]}^{T}$ denote the weight of patch logits $\left[p_{i1},\cdots ,p_{iM}\right]$, respectively. *M* is the number of patches.

To better explain the image-level decision of deep neural networks (DNNs), in this paper, we propose a general attention mechanism to mine significant patches in an image for decision-making, with considering the patches’ spatial relationship yet without using any additional annotations. The proposed attention mechanism can be applied to different deep architectures including convolutional or capsule networks, so that their image-level decision is a weighted sum of patches. Three major contributions of this paper are listed as follows:

- We propose a novel loss-based attention mechanism, namely Loss-Attention, by using the same parameters to learn patch weights and logits (class vectors), and image prediction simultaneously, for connecting the attention mechanism with the loss function. Specifically, the proposed attention mechanism is to mine significant patches and the new loss function is to further boost their precision and recall.
- Based upon Loss-Attention, we propose two deep architectures by modifying current popular CNNs with preserving the spatial relationship of patches in an image for better interpretation, e.g. the image-level decision is a weighted sum of patches. One architecture exploits convolutional layers and the other one adopts capsule layers. For clarity, we present the idea of the proposed convolutional architecture in Fig. 1. The proposed capsule architecture is very similar to Fig. 1 and can be found on released codes.
- Extensive experiments on multiple large-scale benchmark databases demonstrate that the proposed deep architectures can obtain higher or competitive classification accuracy to current popular convoluational or capsule networks, with better interpretable capability. It is worth noting that our proposed capsule architecture can obtain competitive or even better performance than the popular convolutional networks on large-scale complex databases.

## II. Related Work

In this section, we will briefly review some related work including visual interpretability of CNNs, part-based models, capsule networks, and attention-based deep multiple instance learning (MIL).

### A. Visual Interpretability of CNNs

Numerous methods have been proposed to explore visual interpretability of CNNs, including network visualization, model diagnosis, the disentanglement of CNN representations, and explainable models. References [16], [17] are popular network visualization methods, which exhibit the image appearance that maximizes the score of a given unit. Another popular network visualization technique is the up-convolutional net [18], which inverts CNN feature maps into images. Model diagnosis methods [7], [19]–[21] analyze CNN features to visual image regions that contribute the most to the decision-making of CNNs. Disentangling CNN representations is to disentangle complex feature maps in conv-layers into human-interpretable representations. [6], [22] select units from feature maps to describe “scenes” and [23] discovers objects from feature maps of unlabeled images. Reference [24] mines object-part concepts from a pre-trained CNN by extracting certain neural units from feature maps of a filter, with using some object part annotations. Most of aforementioned methods focus on the understanding of a pre-trained CNN, but explainable models aim to learn disentangled representations of neural networks with clear semantic meanings. Reference [25] is a popular interpretable method, which automatically assigns each filter in a high conv-layer with an object part during training. Additionally, visual interpretability methods usually generate class-discriminate representations, fine-grained representations or both. Unlike previous fine-grained approaches [17], [26] learning pixel-space representations, the proposed method is similar to the class-discriminate methods [21], [22], which generate class-discriminative representations. This is because the proposed method learns patch weights by using class information of the corresponding image. Previous methods collect evidence from filters or feature maps to implicitly explain the decision-making of nowadays CNNs, which do not consider the spatial relationship of features. By contrast, the proposed method considers the patches’ spatial relations to directly and explicitly utilize a weighted sum of patches for an image-level decision, and mines the significant patches, which contain objects or their parts determining the image-level prediction.

### B. Part-Based Models

Object parts play a significant role in object recognition, because they are able to capture localized discriminative features of an object. Numerous detection methods are on the basis of object parts. One popular method is deformable part model (DPM) [27], which learns part constellation models with the latent discriminative support vector machine (SVM). However, these methods require ground-truth bounding box annotations. Recently, some CNN-based methods learn or select object parts without any additional part or bounding box annotations. Reference [23] learns part models by finding constellations of neural activation patterns. Reference [28] utilizes elastic non-negative matrix factorization to analyze the response of a pre-trained CNN and extract salient image regions. Reference [29] proposes a multi-attention CNN in order to reinforce part generation and feature learning. These methods are usually on the basis of pre-trained CNNs and most of them cannot directly and explicitly measure the significance of object parts on image-level decision during training. By contrast, the proposed method modifies the architectures of CNNs to preserve the spatial relationship of patches, so that the image-level decision is a weighted sum of patches. And meanwhile it can directly mine significant objects or their parts during training.

### C. Capsule Networks

A capsule is constituted by a group of neurons [9] and thus it outputs an activity vector instead of a scalar to represent different properties of a specific entity, such as an object or its part. Because CNNs cannot preserve the spatial relationship of features by using the pooling layer, e.g. max-pooling, [10] proposes dynamic routing using “routing-by-agreement” between capsules to substitute max-pooling. So it can obtain better performance and more benefits on image interpretation. Reference [30] adopts EM routing for matrix capsules with representing each entity by a pose matrix. Reference [31] formulates dynamic routing as an optimization problem. DeepCaps [11] proposes 3D-convolution-based routing to replace the original dynamic routing for significantly decreasing computation costs. Although capsule networks have achieved promising performance on several popular simple databases and shown strong benefits on image interpretation, their performance on complex databases is still not on a par with that of CNNs. Additionally, the routing strategy can be viewed as an attention mechanism [30], but it is different from the proposed Loss-Attention: (i) The vector outputs of capsules have distinct length in Loss-Attention, while the routing strategy usually squashes the vector outputs of capsules to equal length. This means that Loss-Attention and the routing strategy utilize different ways to calculate the significance of capsules. (ii) Loss-Attention aims to discover the significant patches in an image so that the image-level decision is a weighted sum of patches, but the routing strategy fails to explicitly explore the significance of patches for the image decision-making.

### D. Attention-Based Deep MIL

MIL has been widely applied to real-world applications [32], [33], where only a general statement of the category is given for multiple instances. For example, one bag is composed of tens or hundreds of instances, and it is usually described by a single bag label and there is no label information associated with instances. Although attention mechanisms [12], [34] with DNNs have been successfully used in many tasks, such as image captioning and classification, few efforts focus on attention mechanisms for deep MIL. One popular method is attention-based deep MIL (ADMIL) [13], which proposes two attention mechanisms by using a two-layered neural network to learn instance weights. However, these two attention mechanisms might attain inferior performance to mean-pooling [35] on large-scale image classification in many cases, because they can easily assign large weights to trivial patches. To reduce the effect of trivial patches, loss-based attention mechanism [36] has been proposed to simultaneously learn instance weights and generate bag-level prediction. But its attention mechanism is on the basis of the softmax+cross-entropy function, thereby possibly being ineffective to remove the trivial patches and only suitable for the single-label applications. By contrast, the proposed Loss-Attention is based on the $l_{2,1}$-norm to encourage row-sparsity. It can be applied to both single-label and multi-label scenarios, and simultaneously learn patch weights and logits (class vectors), produce image-level prediction, and remove the trivial patches.

## III. Connecting Attention Mechanism With Loss Function

### A. Preliminaries

We first briefly review two popular loss functions including softmax+cross-entropy and sigmoid+binary-cross-entropy, which will be utilized in the proposed objective for tackling with single-label and multi-label tasks, respectively, and an $l_{2,1}$-norm used in our attention mechanism. For brevity, we introduce the two loss functions using only one training sample.

#### 1) *Softmax*+*Cross-Entropy:*

Given a single-label training sample $X\in {\mathbb{R}}^{C^{0}\times H\times W}$ and its corresponding one-hot label vector $y={\left\{y_{k}\right\}}_{k=1}^{K}\in {\left\{0,\;1\right\}}^{K}$, and an *L*-layer deep neural network $f_{\theta }(\cdot )$ with the parameters ${\left\{\theta^{l}\right\}}_{l=1}^{L}$, where *C*^0^, *H* and *W* denote image channels, height and width, respectively, *K* is the number of classes, and *θ*^*l*^ represents the parameters of the *l*^*th*^-layer in the neural network. Let $z={\left\{z_{k}\right\}}_{k=1}^{K}=f_{\theta }(X)\in {\mathbb{R}}^{K}$ be the output for **X** in the *L*^*th*^ layer of the network, and $s(z)\in {\mathbb{R}}^{K}$ be the estimated class probability of **X**, where s(⋅) denotes the softmax function and ${\text{Σ}}_{k=1}^{K}s\left(z\right)\left[k\right]=1$. To measure the dissimilarity between the true class probability **y** and the estimated class probability *s(***z***)*, the cross-entropy loss is [37]:

(1) $$L_{ce}=-\sum_{k=1}^{K}y_{k}log\left(s(z)[k]\right).$$

Because **X** is a single-label sample and $y\in {\left\{0,1\right\}}^{K}$, we have ${\text{Σ}}_{k=1}^{K}y_{k}=1$. Suppose that **X** belongs to the *t*^*th*^ class, i.e. *y*_*t*_ = 1 and ${\text{Σ}}_{k=1,k\neq t}^{K}y_{k}=0$, Eq. (1) equals:

(2) $$L_{ce}=-log(s(z)[t]).$$

#### 2) *Sigmoid*+*Binary-Cross-Entropy:*

When **X** is a multi-label training sample, because the softmax function is usually suitable for single-label classification tasks and exhibits inferior performance on multi-label applications, $\sigma (z)\in {\left[0,1\right]}^{K}$ is often employed to handle multi-label tasks, where σ(⋅) denotes the sigmoid function. Binary-cross-entropy is defined as:

(3) $$L_{bce}=-\left[y\cdot log(\sigma (z))+\left(1_{K}-y\right)\cdot log\left(1_{K}-\sigma (z)\right)\right].$$

where $1_{K}\in {\mathbb{R}}^{K}$ is a vector with all entries being ones.

#### 3) $l_{2,1}$-Norm:

For a matrix $Z={\left[z_{1},z_{2},\cdots ,z_{N}\right]}^{T}\in {\mathbb{R}}^{N\times K}$, the $l_{2,1}$-norm of **Z** is defined as:

(4) $${∥Z∥}_{2,1}=\sum_{i=1}^{N}\sqrt{\sum_{k=1}^{K}z_{ik}^{2}}.$$

Eq. (4) can encourage the row-sparsity of **Z** [38], [39], because it is the minimum convex hull of the $l_{2,0}$-norm of **Z**, i.e. $∥Z{∥}_{2,0}$, which is to count the number of non-zero rows of **Z**. Additionally, Eq. (4) has the property of rotational invariance [40].

### B. Loss-Based Attention

**Fig. 2.**
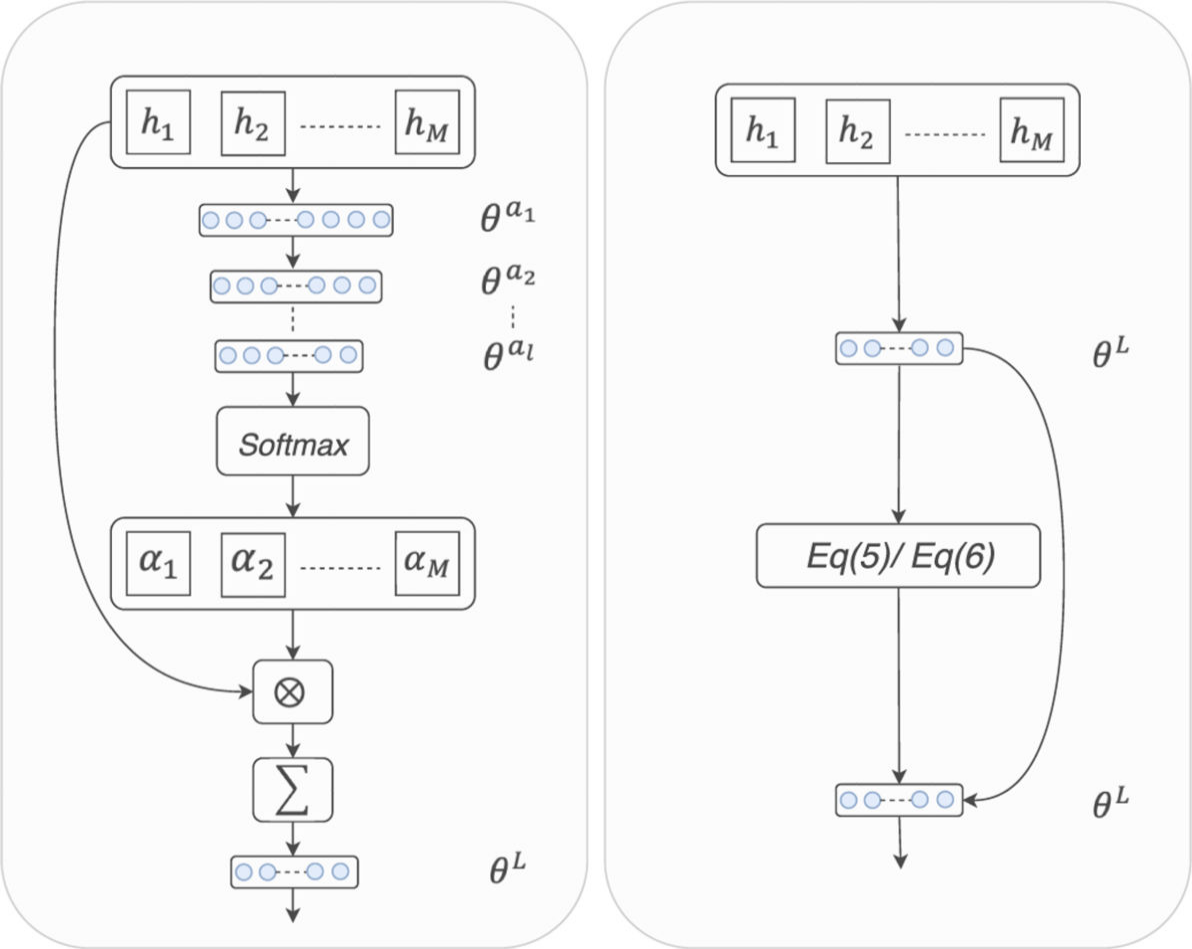
Two different architectures of attention mechanisms. **Left:** Traditional attention mechanism. **Right:** The proposed attention mechanism. $\left[h_{1},h_{2},\cdots ,h_{M}\right]$ represents the feature representation of patches, *θ*^*a*1^, *θ*^*a*2^ and *θ*^*al*^ are the parameters of the attention mechanism for weight generation, $\left[\alpha_{1},\alpha_{2},\cdots ,\alpha_{M}\right]$ is the weight of patches, and *θ*^*L*^ denotes the parameters for image prediction. Note that in the proposed attention mechanism, *θ*^*L*^ is used for both the attention mechanism Eq. (5) or Eq. (6) and image prediction.

Traditional attention mechanisms [13] learn patch weights and image prediction using different layers and parameters, and thus the image classification accuracy is significantly affected by the effectiveness of learned patch weights. To address this issue, we learn the patch weights and logits and generate image prediction simultaneously in order to connect the attention mechanism and the loss function. Specifically, the proposed attention mechanism is on the basis of the $l_{2,1}$-norm [40] and connects with the loss function, i.e. sharing the same parameters with a fully connected layer for image classification and calculating patch weights based on their logits. For clarity, we show the difference between traditional attention mechanisms and the proposed one in Fig. 2. The proposed loss function employs the learned weights to guarantee the selected patches to be within the same class as its image.

#### 1) Attention Mechanisms:

Because convolutional and capsule neural networks are two different architectures, which have distinct outputs for one training sample $X\in {\mathbb{R}}^{C^{0}\times H\times W}$, in the following we present general attention mechanisms for these two different architectures based on their outputs. To avoid the abuse of symbols, we still utilize $f_{\theta }(\cdot )$ to represent the *L*-layer convolutional or capsule neural network.

##### a) Attention for convolutional neural networks:

Suppose that the image **X** is divided into *M* patches, and $H={\left\{h_{m}\right\}}_{m=1}^{M}\in {\mathbb{R}}^{C\times M}$ is its output of the *L*-1^*th*^ layer, and $\theta^{L}\in {\mathbb{R}}^{C\times K}$ denotes the parameters of the *L*^*th*^ layer, where $h_{m}\in {\mathbb{R}}^{C}$ represents the feature representation of the *m*^*th*^ patch of the image **X**, and *C* is the number of channels. Let $P={\left\{p_{m}\right\}}_{m=1}^{M}$ be the *L*^*th*^-layer output for image patches, where $p_{m}\in {\mathbb{R}}^{K}$ is the logit (class vector) for the *m*^*th*^ patch and it is calculated as $p_{m}=h_{m}\theta^{L}$. Then we present the proposed attention mechanism as follows:

(5a) $$\alpha_{j}=\frac{\sqrt{\sum_{k=1}^{K}p_{jk}^{2}}}{\sum_{m=1}^{M}\sqrt{\sum_{k=1}^{K}p_{mk}^{2}}},$$

(5b) $$\alpha_{j}\leftarrow \frac{max\left(\alpha_{j}-\frac{\xi }{M},0\right)}{\sum_{m=1}^{M}max\left(\alpha_{m}-\frac{\xi }{M},0\right)},$$

(5c) $$h_{j}\leftarrow \alpha_{j}h_{j},$$

(5d) $$z=\sum_{m=1}^{M}h_{m}\theta^{L},$$

where *α*_*j*_ is the attention weight of the *j*^*th*^ patch of X,ξ∈[0,1] is a threshold to remove the trivial patches, and $z\in {\mathbb{R}}^{K}$ is the *L*^*th*^-layer output for **X**. It is worth noting that Eq. (5a) utilizes the $l_{2,1}$-norm, i.e. $\sum_{m=1}^{M}\sqrt{\sum_{k=1}^{K}p_{mk}^{2}}$, to encourage the row-sparsity of $P\in {\mathbb{R}}^{M\times K}$, so as to enhance the weights of significant patches and decrease the weights of trivial patches. Additionally, we empirically set the maximum of ξ as 1 during the training process, because all patch weights might be zeros during training when ξ>1.

##### b) Attention for capsule neural networks:

Suppose that $H={\left\{H_{m}\right\}}_{m=1}^{M}\in {\mathbb{R}}^{C\times M\times D}$ is the output of the *L*-1^*th*^ layer of a capsule network for **X,** where $H_{m}={\left\{h_{cm}\right\}}_{c=1}^{C}\in {\mathbb{R}}^{C\times D}$ represents the feature representation of the *m*^*th*^ patch for the image **X**, *C* is the number of channels and *D* is the capsule dimension. Let $\theta^{L}\in {\mathbb{R}}^{D\times K}$ denote the parameters of the *L*^*th*^ layer, and **P** be the *L*^*th*^-layer output corresponding to **H**, e.g. $P_{m}={\left\{p_{cm}\right\}}_{c=1}^{C}$, be the *L*^*th*^-layer output corresponding to $H_{m}$, where $p_{cm}=h_{cm}\theta^{L}\in {\mathbb{R}}^{K}$. Afterward, we introduce the proposed attention mechanism as follows:

(6a) $$\alpha_{rj}=\frac{\sqrt{\sum_{k=1}^{K}p_{rjk}^{2}}}{\sum_{c=1}^{C}\sum_{m=1}^{M}\sqrt{\sum_{k=1}^{K}p_{cmk}^{2}}},$$

(6b) $$\alpha_{j}=\frac{max\left(\sum_{c=1}^{C}\alpha_{cj}-\frac{\xi }{M},0\right)}{\sum_{m=1}^{M}max\left(\sum_{c=1}^{C}\alpha_{cm}-\frac{\xi }{M},0\right)},$$

(6c) $$\alpha_{rj}\leftarrow \frac{sgn\left(\alpha_{j}\right)\alpha_{rj}}{\sum_{c=1}^{C}\sum_{m=1}^{M}sgn\left(\alpha_{m}\right)\alpha_{cm}},$$

(6d) $$h_{rj}\leftarrow \alpha_{rj}h_{rj},$$

(6e) $$z=\sum_{m=1}^{M}\sum_{c=1}^{C}h_{cm}\theta^{L},$$

where *α*_*rj*_ denotes the attention weight of the *j*^*th*^ patch of **X** at the *r*^*th*^ channel, sgn(⋅) is a function defined as: *sgn*(*α*_*m*_) = 0 if *α*_*m*_ = 0, and *sgn*(*α*_*m*_) = 1 when *α*_*m*_ *>* 0.

#### 2) Loss Function via Attention Weights:

Based on the attention mechanism Eq. (5) or (6), we can obtain the weight of each image patch. However, when directly utilizing the loss in either Eq. (2) or Eq. (3) for model training, it might have two issues: (i) a trivial patch with a large weight, although *ξ* can remove some trivial patches; (ii) low significant patch recall. For better illustrating these two issues, based on the output of convolutional networks for the sample **X**, we present two propositions as follows. Their detailed proofs are shown in the Appendix.

##### Proposition 1:

*For an image* **X** *with M patches, suppose that* $q_{mt}=\frac{e^{p_{mt}}}{\sum_{k=1}^{K}e^{p_{mk}}}$ *denotes the estimated class probability of the m^th^ patch belonging to the t^th^ class. For the objective of Eq. (2), there exists*:

(7) $$L_{ce}\geq \frac{\sum_{k=1,k\neq t}^{K}\prod_{m=1}^{M}{\left(\frac{q_{mk}}{q_{mt}}\right)}^{\alpha_{m}}}{1+\sum_{k=1,k\neq t}^{K}\prod_{m=1}^{M}{\left(\frac{q_{mk}}{q_{mt}}\right)}^{\alpha_{m}}}.$$

##### Proposition 2:

*For an image* **X** *with M patches, a lower bound of the objective Eq. (3) is*:

(8) $$L_{bce}\geq \sum_{k=1,y_{k}=1}^{K}\frac{\prod_{m=1}^{M}{\left(e^{-p_{mk}}\right)}^{\alpha_{m}}}{1+\prod_{m=1}^{M}{\left(e^{-p_{mk}}\right)}^{\alpha_{m}}}\;+\sum_{k=1,y_{k}=0}^{K}\frac{1}{1+\prod_{m=1}^{M}{\left(e^{-p_{mk}}\right)}^{\alpha_{m}}}.$$

Eq. (7) suggests that when $L_{ce}\to 0$, at least one patch of the image **X** belongs to the *t*^*th*^ class. Specifically, for any patch, if it has $\frac{q_{mk}}{q_{mt}}\to 0\;(\forall k\neq t)$ and $\alpha_{m}\gg 0$, then $L_{ce}\to 0$. However, $L_{ce}\to 0$ cannot theoretically guarantee the patch with a large weight and more than one patch assigned to the *t*^*th*^ class, thereby potentially assigning a large weight to a trivial patch and leading to the low significant patch recall. For Eq. (8), when $L_{bce}\to 0$, at least one significant positive image patch and one negative patch will be assigned weights larger than zeros. Unfortunately, it still cannot guarantee more than one positive or negative significant patch to be selected, and it is also very likely to assign a large weight to a trivial patch. Similar findings can be obtained from the attention mechanism for capsule networks.

To alleviate the aforementioned two issues, based on Eqs. (2) and (3), we introduce regularization terms using the weights obtained from the proposed attention mechanism Eq. (5) and (6), and present the following loss functions to handle single-label and multi-label tasks, respectively. Specifically, given training data $\text{Ψ}={\left\{X_{i}\right\}}_{i=1}^{N}$, let *B* denote the index set of selected training samples in each mini-batch, y_*i*_ be the one-hot label vector of **X**_*i*_ and **z**_*i*_ represent its *L*^*th*^-layer output in convolutional or capsule neural networks. The proposed loss function for single-label tasks is:

(9) $$L_{s}=-\frac{1}{\left|B\right|}\sum_{i\in B}[\sum_{k=1,y_{ik=1}}^{K}log\left(s\left(z_{i}\right)\left[k\right]\right)+\gamma \left(\tau \right)\sum_{m=1}^{M}\alpha_{im}\sum_{k=1,y_{ik}=1}^{K}log\left(s\left(p_{im}\right)\left[k\right]\right),$$

where |B| denotes the number of selected images in the mini-batch, the regularization term is to enforce selected patches to share the same class with the image, γ(τ) is an unsupervised weighting function to balance the weight between image and patch classification, and *τ* is the number of current training epochs.

Based on Eq. (3), the proposed loss function for multi-label tasks is:

(10) $$L_{m}=-\frac{1}{\left|B\right|}\sum_{i\in B}\left[y_{i}\cdot log\left(\sigma \left(z_{i}\right)\right)+\left(1_{K}-y_{i}\right)\cdot log\left(1_{K}-\sigma \left(z_{i}\right)\right)+\gamma \left(\tau \right)\sum_{m=1}^{M}\alpha_{im}\underset{1\leq k\leq K,y_{ik}=1}{max}log\left(\sigma \left(p_{im}\left[k\right]\right)\right)\right],$$

where the term $\underset{1\leq k\leq K,y_{ik}=1}{max}log\left(\sigma \left(p_{im}[k]\right)\right)$ aims to make the selected patch share at least one class label with the image **X**_*i*_. Thus, the proposed method can discover significant patches while ignore trivial ones.

## IV. Network Architectures

**Fig. 3.**
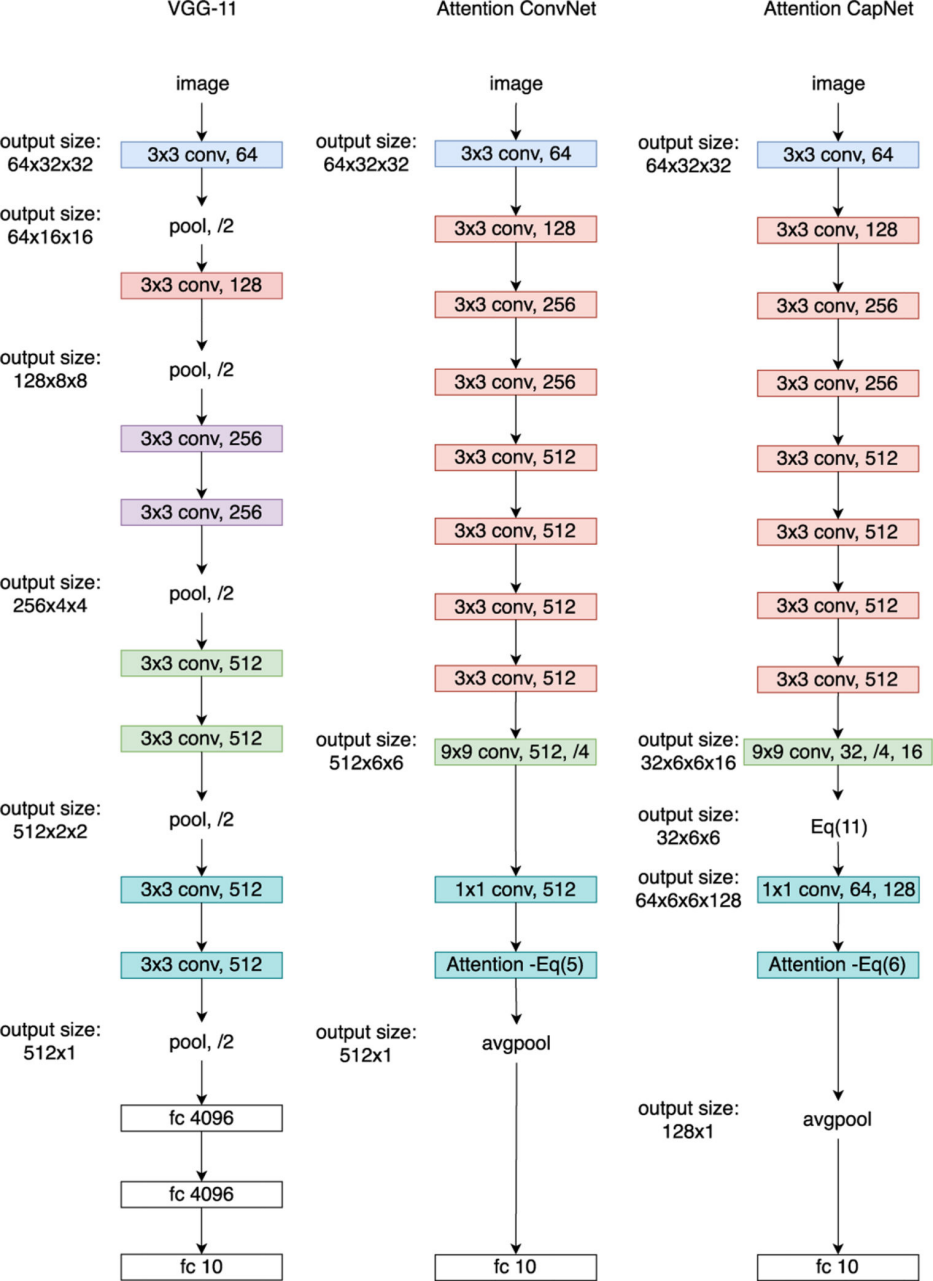
The architecture of different networks for CIFAR-10. **Left:** the VGG-11 model as a reference. **Middle:** a convolutional architecture with the attention mechanism Eq. (5). **Right:** a capsule architecture with the attention mechanism Eq. (6).

Most current CNNs do not preserve the spatial relationship of features in one image. This is because they usually adopt max-pooling or stride operations following by large convolution kernels (whose size is larger than 1), and thus the effect of any part of the input on a hidden activation depends on other parts. Additionally, the activity of one hidden unit depends on the activity of other hidden units [41]. These two causes significantly increase the difficulty to interpret CNNs. To maintain the spatial relationship of patches and reduce the complex dependency between input and hidden activations for better interpretation, e.g. the image-level decision is a weighted sum of patches, we propose two schemes (one with convolutional layers and the other using capsule layers) by modifying CNNs. In the following, we present the two schemes based on one popular network, VGG-11 [14] (The left architecture of Fig. 3). Modification on other network, such as ResNet, is similar. Due to limited space, we provide more details on released codes.

### A. Convolutional Architecture

We first remove the max-pooling operations and two fully-connected layers in VGG-11, to preserve the spatial relationship of patches within an image and reduce the complex dependency between input and hidden activations. Next, we introduce one convolutional layer with 512 channels, kernel size 9 × 9 and stride 4 to determine the size and number of patches and extract their features, and another convolutional layer with 512 channels, kernel size 1 × 1 and stride 1 for the nonlinear mapping of patch features. The 1 × 1 kernel is to reduce the dependency among patch features. Then we add an attention layer using Eq. (5) to select significant patches based on the attained patch features. For clarity, we present this architecture in the middle part of Fig. 3.

### B. Capsule Architecture

We first remove the max-pooling and two fully-connected layers in VGG-11. Then we add two capsule layers, including one capsule with 32 channels, kernel size 9 × 9, stride 4, and capsule dimension 16, and the second capsule with 64 channels, kernel size 1 × 1, stride 1 and capsule dimension 128, so that the capsule architecture has the very similar number of parameters to the convolutional one. To better show the interaction between two capsule layers, we use an example image for illustration as follows:

For an image $X\in {\mathbb{R}}^{3\times 32\times 32}$, let $\text{let }\tilde{H}={\left\{{\tilde{H}}_{d}\right\}}_{d=1}^{16}\in {\mathbb{R}}^{32\times 6\times 6\times 16}$ be the output of the first capsule layer. We trans form $\tilde{H}$ into the following form:

(11) $$\tilde{H}\leftarrow max(\sqrt{\sum_{d=1}^{16}{\tilde{H}}_{d}}-b1_{1\times 6\times 6},0),$$

where ${\tilde{H}}_{d}\in {\mathbb{R}}^{32\times 6\times 6},b\in {\mathbb{R}}^{32}$ is to remove trivial image pixels in each channel, and $1_{1\times 6\times 6}\in {\mathbb{R}}^{1\times 6\times 6}$ is a matrix with all entries being ones in order to expand **b** to have the same size as ${\tilde{H}}_{d}$ Based on Eq. (11), $\tilde{H}\in {\mathbb{R}}^{32\times 6\times 6}$ will be fed into the second capsule layer. Afterward, we adopt an attention layer using Eq. (6) to assign a weight to each capsule and select significant patches. For better illustration, we present this capsule architecture in the right part of Fig. 3.

The size of input images is 32 × 32 in Fig. 3. When input images have a larger size, they will consume much more computation and memory costs. In this case, we can utilize stride operations in convolutional layers of the backbone network only to reduce the image size, and then adopt the proposed two convolutional or capsule layers and the attention layer to preserve their spatial relationship and discover the significant patches. Note that we do not adopt max-pooling to reduce the image size, because it might lose some useful information. Moreover, the proposed schemes can also be applied to other CNNs, such as ResNet [5], upon which we can first remove the stride operations in convolutional layers, and then add the proposed convolutional or capsule and attention layers. In addition to VGG-11, in our experiments we apply the proposed two schemes on a popular network ResNet18.

## V. Experimental Results and Analysis

To evaluate the proposed architectures, we conduct experiments on multiple large-scale benchmark databases for image classification and patch interpretability.

### A. Implementation Details

We implement the proposed architectures by using the PyTorch framework and adopt VGG11_bn [14] and ResNet18 [5] as our backbone networks mostly. We employ the optimizer, SGD, to update model parameters, and totally run the model 200 epochs with a batch size being 128. By default, we first train the model 100 epochs using the learning rate *η* = 0.1, and then run the model 50 epochs via the learning rate 0.01, afterwards, we set the learning rate to 0.001 during the last 50 epochs. For the threshold *ξ* in Eqs. (5)-(6), we set ξ=0.1 in default. For the unsupervised weighting function γ(τ), we utilize a Gaussian ramp-up function $\gamma_{max}e^{-∥1-T{∥}_{F}^{2}}$ and set $\gamma_{max}=0.1$, where *T* linearly increases from 0 to 1 during the first 80 epochs, and then it keeps unchanged.

### B. Experimental Settings

Because the proposed architectures utilize VGG11_bn and ResNet18 as their backbone networks, we compare them with the baseline methods VGG11_bn and ResNet18. Additionally, because the proposed method adds convolutional layers, which might increase model parameters, for a fair and better comparison, we report the classification results of VGG16_bn and ResNet50, which have more parameters than our convolutional architectures. Moreover, to better illustrate the strength of the proposed Loss-Attention, we present the results of Mean-pooling, Attention and Gated-Attention [13], and Dynamic Routing [10] using our modified architectures. Mean-pooling means assigning each patch to the same weight. Note that Attention and Gate-Attention utilize the same training procedure as our method, but Mean-pooling and Dynamic Routing do not exploit this procedure. Thus, we adopt a different learning procedure for Mean-pooling and Dynamic Routing as follows: we adopt the optimizer, Adam [42], with initializing momentum parameters *β*_1_ = 0.9 and *β*_2_ = 0.99. We also train the model 200 epochs. The learning rate ramps up to the maximum 0.003 during the first 80 epochs by using the function $e^{-∥1-T{∥}_{F}^{2}}$. Then the learning rate keeps unchanged during the following 40 epochs; afterward, the learning rate decreases to 0.0003, and it becomes 0.00003 during the last 40 epochs. The Adam momentum parameter *β*_2_ becomes 0.999 after the first 80 epochs. We run each experiment 4 times and calculate the average accuracy. Note that for the proposed method, the selection of batch size, optimizer type, learning rate and its strategy is the same as the backbone network. However, the performance of Mean-pooling and Dynamic Routing might be greatly affected by different optimizers, e.g., Adam and SGD (see Table I). The major possible reason is that Mean-pooling and Dynamic Routing cannot provide significant patches, so that trivial patches significantly affect the gradient update. Additionally, Adam can be viewed through the lens of clipping, thereby leading to better performance in heavy-tail noise settings [43].

### C. Experiments for Image Classification

We run experiments to evaluate the proposed architectures on image classification by using the following popular single-label databases:

**CIFAR-10** [45] consists of 60K color images in 10 classes, each of which contains 6K images. These images are divided into a training set of 50K examples and a testing set of 10K ones. Each one is aligned and cropped to 32 × 32 pixels.

**CIFAR-100** [45] is composed of 60K color images belonging to 100 classes, with 600 images per class. These images are also divided into 50K training and 10K testing ones. Each image is with a size of 32 × 32.

**SVHN** [46] has 73,257 training, 26,032 testing and 531,131 additional digits, which are from ‘0’ to ‘9’. Each digit is cropped and resized to 32 × 32. We adopt 73,257 training and 26,032 testing digits in our experiments.

#### 1) Experimental Results:

On the three databases, Loss-Attention adopts Eq. (9) for classification. Besides the four comparative methods, Mean-pooling, Attention and Gated-Attention, and Dynamic Routing, we also present the results of several popular capsule networks [10], [11], [47], [48] to better evaluate the proposed capsule architecture.

Table II presents the classification accuracy of different deep methods. For convolutional networks, when using VGG11_bn as the backbone network, Mean-pooling, Attention, Gated-Attention and Loss-attention obtain superior performance over VGG11_bn and VGG16_bn on CIFAR-10 and CIFAR-100, and Loss-Attention achieves better classification accuracy than the other methods on all the three databases. Additionally, when using ResNet18 as the backbone network, Loss-Attention also attains better accuracy than the others on CIFAR-10 and CIFAR-100, and achieves competitive performance with the best competitors on SVHN. These results suggest that the proposed architectures, whose image-level decision is a weighted sum of patches, can obtain better or competitive classification performance with popular CNNs.

For capsule networks, Loss-Attention obtains superior performance over Dynamic Routing and other deep capsule methods [10], [11], [47], [48] when using VGG11_bn and ResNet18 as backbone networks. Moreover, Loss-Attention with the capsule architecture can achieve competitive and even better classification accuracy than that with the convolutional architecture on CIFAR-10 and SVHN. The capsule architecture attains slightly worse accuracy than that with the convolutional one on CIFAR-100, probably because its capsule dimension is similar to the number of classes. They suggest that capsule networks with Loss-Attention can obtain superior or similar performance to convolutional ones on complex databases. It is worth noting that when using our proposed architecture with VGG11_bn and ResNet18 as backbone networks, Dynamic Routing can attain better performance than the deep capsule methods [10], [11], [47], [48] on CIFAR-10, and it only attains slightly worse accuracy than DeepCaps on SVHN.

The proposed method can also be applied to more deeper versions of ResNet or other different architectures. Table III displays the accuracy of Loss-Attention with ResNet50 and GoogleNet [15] as backbone networks on CIFAR-10 and CIFAR-100. It suggests that Loss-Attention outperforms Baseline (ResNet50 and GoogleNet). Additionally, Loss-Attention can achieve better performance on large-scale databases. For example, when using ResNet18 as the backbone, the accuracy of Loss-Attention and ResNet18 is 56.57% and 55.40% respectively on ImageNet [2], where each image is resized to 32 × 32. Additionally, Loss-Attention using ResNet18 as the backbone takes one week to train a model for ImageNet, with 4 GPUs and a batch size being 128. When we utilize ResNet50 and GoogleNet as the backbone, the time cost for model training is respectively 4.5 and 6 times more than using ResNet18. Hence, here we do not show their results on ImageNet because of limited resources and spaces.

### D. Experiments for Image Patch Interpretability

Because test images in the aforementioned databases do not contain bounding boxes, we run experiments for image patch interpretability on two popular databases with bounding boxes as follows:

**Tiny ImageNet** [49] is a single-label database, which has 200 classes with each category consisting of 500 training, 50 validation and 50 test images. Among them, validation and test images have bounding boxes. We adopt training images as a training set and validation images for test. Each image is with a size of 64 × 64.

**Microsoft COCO** [50] is one multi-label database, which consists of around 328,000 images belonging to 91 object types. We utilize the 2014 training and validation sets, including 82,081 training and 40,137 validation images. We adopt the training images for training and validation ones for testing. We crop and resize each image to 64 × 64 pixels.

Note that we do not resize each image to 32 × 32 in order to illustrate that the proposed architecture can handle a larger image size (*>* 32 × 32).

#### 1) Experimental Settings:

Because the size of images in the two databases is 64 × 64, we adopt stride 2 in the fourth convolutional layer of VGG11_bn and in the sixth layer of ResNet18 and remove max-pooling or stride operations in other layers. The patch sharing at least one common label as its corresponding image and more than half size locating in the bounding box is viewed as a correct one. Additionally, we show the image localization accuracy of Attention, Gated-Attention and Loss-Attention on Tiny ImageNet by using the estimated bounding box, which is the minimum square to contain selected patches. For Loss-Attention, we select the patches with weights larger than 0, and for Attention and Gated-Attention, we choose the patches with weights bigger than $\frac{\xi }{M}$. The estimated bounding box is considered correct if intersection over union (IoU) is larger than 0.5. Then we show the average precision (AP) of image localization. Moreover, we present the image classification accuracy (Accuracy for Tiny ImageNet and mAP for COCO, where mAP is defined in [44]) of the aforementioned methods and the baselines VGG11_bn and ResNet18. Note that we do not report the performance of Dynamic Routing on Tiny ImageNet due to its high memory cost for a large number of classes. We also do not show the image localization accuracy AP of COCO, because many images contain multiple bounding boxes belonging to one category of objects and the attention methods cannot directly handle this case. For Loss-Attention, we utilize the aforementioned parameter settings for image classification, and we adopt Eq. (9) for Tiny ImageNet and Eq. (10) for COCO to train models.

#### 2) Experimental Results:

Tables IV-V present the performance of different deep methods on Tiny ImageNet and COCO. Attention and Gated-Attention obtain better image classification accuracy, patch precision and recall than Mean-Pooling on Tiny ImageNet, while they achieve significantly worse performance than Mean-Pooling on COCO. This might be because they align large weights to trivial patches and obtain low patch recall, thereby decreasing the model performance. Loss-Attention obtains better image classification and localization accuracy, and F-score for patches than Mean-pooling, Attention, Gated-Attention on the two databases. The proposed attention mechanism can remove trivial patches, and the introduced regularization term in the loss function can further boost the patch precision and recall, thereby decreasing the effect of trivial patches on model performance. Loss-Attention with the modified convolutional architecture also outperforms the baseline methods on image classification. For example, when using VGG11_bn as the backbone network of convolutional architectures, Loss-Attention attains 0.83% higher image classification accuracy, 1.78% better AP and 13.27% F-score than the best competitors on Tiny ImageNet. It achieves 7.70% higher mAP and 1.70% F-score than the best competitors on COCO. Additionally, Loss-Attention achieves better image classification and patch precision than Dynamic Routing on COCO. Moreover, Loss-Attention with a convolutional architecture achieves better image classification than that with a capsule architecture on Tiny ImageNet and COCO. This might be because the capsule architecture adopts a small capsule dimension, which is less or close to the number of classes on the two databases.

**Fig. 4.**
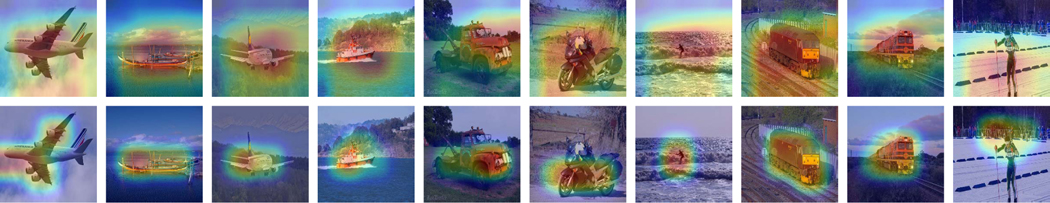
Heat maps of sample images from COCO by using Grad-CAM [21] and the convolutional architecture+Loss-Attention. Both of them adopt ResNet18 as the backbone network. The first and second rows show heat maps of Grad-CAM and Loss-Attention, respectively.

**Fig. 5.**
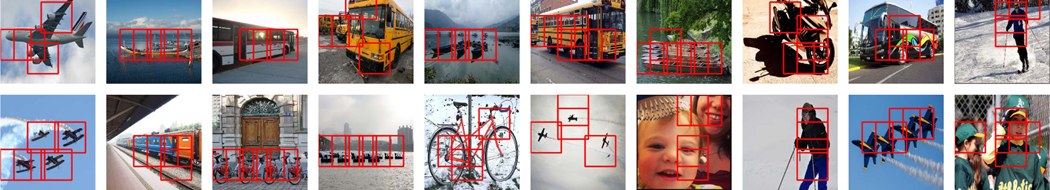
Selected patches of some images from COCO by using the convolutional architecture+Loss-Attention with ResNet18 as the backbone network.

**Fig. 6.**
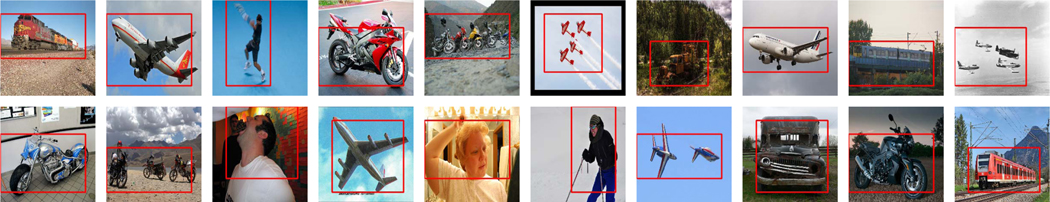
Predicted bounding boxes of some images from COCO by using the convolutional architecture+Loss-Attention with ResNet18 as a backbone network.

To better illustrate the effectiveness of the proposed architectures, Fig. 4 displays heat maps of sample images from COCO by using Grad-CAM [21] and the convolutional architecture+Loss-Attention with ResNet18 as the backbone. It suggests that both Grad-CAM and Loss-Attention can generate class-discriminative representations, but Loss-Attention produces more accurate representations. This is because Loss-Attention selects significant patches and meanwhile removes trivial patches. For clarity, Fig. 5 presents selected patches of some images from COCO by using the convolutional architecture+Loss-Attention with ResNet18. Fig. 6 presents the estimated bounding boxes of some images from COCO with the convolutional architecture. Similar observations can be found when using VGG11_bn as the backbone network or the capsule architecture. They suggest that the proposed architectures can be viewed as a weighed sum of patches, and Loss-Attention can effectively mine the significant patches containing objects or their parts to interpret the image-level decision, i.e. which parts of the image determine the decision-making.

### E. Ablation Study and Parameter Analysis

Here, we evaluate the essential parameters $\gamma_{max}$ and ξ in the proposed Loss-Attention, with the convolutional architecture using VGG11_bn and ResNet18 as backbone networks on Tiny ImageNet. Table VI presents the results of Loss-Attention on setting $\gamma_{max}=0$ or ξ=0. It displays that when $\gamma_{max}=0.1$, ξ=0.1 achieves higher patch precision yet lower recall than ξ=0; when ξ=0.1, $\gamma_{max}=0.1$ attains better image classification and localization, patch precision and recall than $\gamma_{max}=0$. Similar findings can be observed on other databases.

**Fig. 7.**
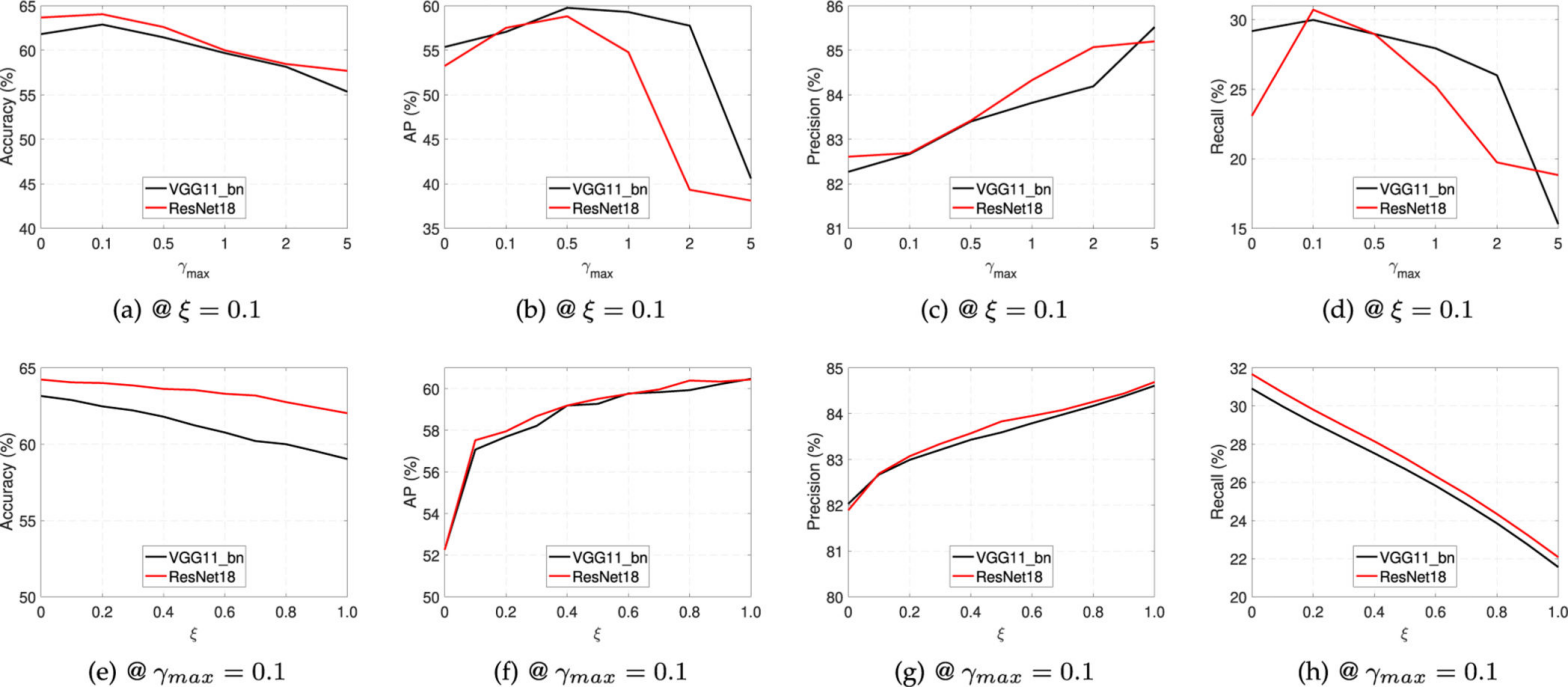
The effect of the parameters $\gamma_{max}$ and *ξ* in Loss-Attention with the convolutional architecture using VGG11_bn and ResNet18 as backbone networks on Tiny ImageNet.

Fig. 7 presents the effects of $\gamma_{max}$ within [0,5] and ξ during [0,1] on Loss-Attention. Fig. 7(a)-(d) show that when ξ=0.1, Loss-Attention attains the best image classification accuracy for $\gamma_{max}=0.1$ and it achieves the best AP for $\gamma_{max}=0.5$, after which the accuracy decreases with the increasing value of $\gamma_{max}$. Patch recall has a similar trend to the image classification accuracy, while patch precision gradually grows with the increasing value of $\gamma_{max}$. They suggest that $\gamma_{max}$ can increase the patch precision when $\gamma_{max}\in [0,5]$, and it can boost the image classification accuracy and patch recall when $\gamma_{max}\in [0,0.1]$, and improve the localization accuracy when $\gamma_{max}\in [0,0.5]$. Fig. 7(e)-(h) illustrate that when $\gamma_{max}=0.1$, AP and patch precision grow with the increasing value of ξ, while the image classification accuracy and patch recall decrease. Similar findings can be observed on COCO, so we do not show them for brevity.

Both Table VI and Fig. 7 infer that the regularization term can be used to boost the image classification and localization accuracy, patch precision and recall. Additionally, ξ can be used to adjust the value of image classification accuracy, AP, patch precision and recall.

### F. Discussion and Analysis

Based on experimental results of image classification in Tables II–V, we can see that the proposed convolutional and capsule architectures significantly reduce the dependency of multiple parts of the input by removing max-pooling or stride operations, so that their image-level decision is a weighted sum of patches. However, they still can achieve competitive and even better performance than the popular CNNs, VGG11_bn, VGG16_bn, ResNet18, ResNet50 and GoogleNet. This is mainly attributed to the loss-based attention mechanism, which can effectively mine the significant image patches. As shown in Tables II–V, Mean-pooling, Attention and Gated-Attention mechanisms cannot always outperform the backbone when they adopt the same backbone networks, but the loss-based attention mechanism usually has superior performance over all of them.

Table II presents that Dynamic Routing with the modified capsule architecture outperforms previous capsule networks on CIFAR-10 and achieves competitive performance to the best competitor on SVHN. This might be because we adopt Adam for Dynamic routing to handle trivial patches [43] and a different training procedure, i.e. gradually increasing the learning rate to smooth the training process, which is usually able to improve the model generalization performance [51]. Additionally, when we utilize the same training procedure as that of previous capsule networks, Dynamic Routing with the modified capsule architecture usually achieves much worse accuracy. They might suggest that previous capsule networks can achieve better performance by using the same procedure as ours. Moreover, the capsule networks with Loss-Attention can achieve better or competitive performance to convolutional networks on CIFAR-10, CIFAR-100 and SVHN. This infer that the performance of capsule networks can be on a par with that of CNNs on complex databases.

Experiments for image patch interpretability (Tables IV-V) suggest that a better patch precision or recall does not always result in a higher image classification or localization accuracy for the proposed convolutional architectures. This is because the image-level prediction is determined by a weighted sum of patches, i.e. each patch has different significance, while the patch precision or recall only shows how many significant patches are selected and does not consider their significance. Therefore, a single patch precision or recall is not correlated with classification and localization. However, as shown in Tables IV-V, a better F-score usually leads to better classification and localization performance for the proposed convolutional architectures on Tiny ImageNet and COCO. Ablation study demonstrates that the parameter *ξ* can remove trivial patches to improve the image localization accuracy and patch precision, and the introduced regularization term can further boost patch precision and recall.

The modified deep architectures consider the spatial relationship of features, and obtain competitive or even higher accuracy than baseline networks with better interpretability. However, because of removing max-pooling or stride operations, they have two major disadvantages: (i) consuming more GPU memory, (ii) increasing computational costs. These are caused by feeding inputs with a larger size into the next layer and using more parameters, e.g. the layer with 512 channels, kernel size 9 × 9 and stride 4 in Fig. 3. In practice, when the image size is larger than 32 × 32, we can add stride into several layers before the two introduced convolutional layers to reduce the image size. For clarity, Table VII presents the classification accuracy and training time of Loss-Attention with ResNet18 on CIFAR-10 for a larger size 64×64. It illustrates that Loss-Attention using one layer with stride 2 consumes less time cost. Additionally, adding stride 2 into one layer achieves higher accuracy than that without using stride. This is because a larger image size usually generates more patches, which increase the difficulty of mining significant patches. Loss-Attention’s time cost mainly depends on the number of layers with stride 2 in the backbone, because the stride can reduce the input size. Meanwhile, the accuracy is very close when the stride is used in the backbone. Moreover, the added convolutional layer using stride 2 only consumes slightly more time than that using stride 4, but with almost the same accuracy. We respectively set kernel size and stride as 9×9 and 4 in our experiments, because we follow the setting in Dynamic Routing for a fair comparison and better interpreting image-level decision. In practice, if we only want to obtain better accuracy than the backbone with low computational complexity, the stride can be used in more convolutional layers.

## VI. Conclusion and Future Work

In this paper, we propose a general attention mechanism and modify previous convolutional and capsule networks to mine significant patches, which contain objects or their parts determining the image-level prediction. The proposed Loss-Attention shares the parameters between attention mechanisms and loss functions to learn patch weights and logits, and image prediction simultaneously, in order to connect the attention mechanism and the loss function for boosting patch precision and recall. The modified deep architectures consider the spatial relationship of features by removing max-pooling or stride operations in convolutional layers, so that the image-level decision is a weighed sum of patches. Extensive experiments on multiple large-scale benchmark databases demonstrate the superior performance of the proposed deep architectures over comparative popular deep neural networks with better interpretation.

Although the proposed architectures can attain promising performance on single-label image localization, it still cannot locate multiple objects belonging to one category in an image. This might be because our method focuses on the patch interpretation rather than region proposal selection. However, it is promising to extend and apply our method for weakly supervised localization on universal scenarios. Additionally, our capsule architecture utilizes convolutional layers as backbone, and in the future it is promising to design different capsule networks based on the proposed two capsule layers to handle large-scale tasks.

## Figures and Tables

**TABLE I:** The Performance (%) of Dynamic Routing and Mean-Pooling Using SGD and Adam on Different Databases, With Employing ResNet18 as the Backbone

Optimizer	Dynamic Routing			Mean-pooling
	Accuracy		mAP [44]	
	CIFAR-10	CIFAR-100	COCO	COCO

SGD	91.82	47.37	22.01	43.02
Adam	**93.88**	**68.88**	**48.19**	**51.43**

**TABLE II:** Classification Accuracy (%) of Two Architectures: Convolutional and Capsule Networks, on Three Large-Scale Benchmark Databases. We Bold the Best Results of Each Architecture and Their Similar Accuracy Within 0.1%.

Backbone	Method	Params (×10^6^)	CIFAR-10	CIFAR-100	SVHN

	ConvNet				

	VGG11_bn	28.15	92.14	70.20	96.48
	VGG16_bn	33.65	93.52	72.35	96.83

VGG11_bn	Mean-pooling	30.73	93.99	74.03	96.37
	Attention	30.80	94.17	75.47	96.52
	Gated-Attention	30.86	93.78	74.35	96.88
	**Loss-Attention**	30.73	94.51	76.81	96.99

	ResNetl8	11.17	94.76	76.61	96.98
	ResNet50	58.16	95.01	78.15	**97.12**

ResNetl8	Mean-pooling	32.67	94.59	75.58	96.72
	Attention	32.73	94.83	77.48	**97.08**
	Gated-Attention	32.80	94.89	76.33	**97.07**
	**Loss-Attention**	32.67	**95.31**	**78.53**	**97.11**

	CapNet				

	Sabour et al. [10]*	-	89.40	-	95.70
	Nair et al. [47]	-	67.53	-	91.06
	HitNet et al. [48]	-	73.30	-	94.50
	DeepCaps [11]^†^	-	91.01	-	**97.16**

VGG11_bn	Dynamic Routing	33.41	93.37	68.41	96.08
	**Loss-Attention**	31.02	94.98	75.84	96.78

ResNetl8	Dynamic Routing	35.35	93.88	68.88	96.70
	**Loss-Attention**	32.96	**95.47**	**76.70**	**97.24**

∗ Denotes the Results Obtained by Ensemble and

† Means the Accuracy Achieved by Using the Image Size 64 × 64

**TABLE III:** Classification Accuracy (%) of Loss-Attention With ResNet50 and GoogleNet as the Backbone on Benchmark Databases. Baseline Denotes the Backbone Network. We Bold the Best Results in Each Setting

Method	ResNet50		GoogleNet	

	CIFAR-10	CIFAR-100	CIFAR-10	CIFAR-100

Baseline	95.01	78.15	94.58	77.45
Loss-Attention	**95.70**	**79.17**	**95.63**	**78.68**

**TABLE IV:** Results (%) of Different Convolutional and Capsule Networks on a Single-Label Database Tiny ImageNet. We Bold the Best Image Classification, Localization Accuracy and F-Score for Patches at Each Group

Method	VGGll_bn					ResNetl8				
	Image		Patch			Image		Patch		
	Accuracy	AP	Precision	Recall	F-score	Accuracy	AP	Precision	Recall	F-score

ConvNet										

Baseline	56.52	-	-	-	-	63.84	-	-	-	-
Mean-pooling	57.83	-	81.85	13.53	23.22	58.79	-	81.67	14.06	23.99
Attention	61.75	55.29	83.88	18.81	30.73	63.22	55.31	84.18	19.24	31.32
Gated-Attentton	60.56	55.29	84.09	16.07	26.98	61.82	55.53	84.12	17.33	28.74
**Loss-Attention**	**62.58**	**57.07**	82.67	29.98	**44.00**	**64.05**	**57.52**	82.69	30.71	**44.79**

CapNet										

**Loss-Attention**	59.58	52.80	81.20	29.98	43.79	61.58	52.96	81.35	32.41	46.35

**TABLE V:** Results (%) of Different Convolutional and Capsule Networks on a Multi-Label Database COCO. We Bold the Best Image Classification Accuracy and F-Score for Patches at Each Group

Method	VGG11_bn				ResNet18			
	Image	Patch			Image	Patch		

	mAP	Precision	Recall	F-score	mAP	Precision	Recall	F-score

ConvNet								

Baseline	40.58	-	-	-	49.35	-	-	-
Mean-pooling	50.16	23.63	69.39	35.25	51.43	23.88	73.10	36.00
Attention	36.70	20.75	48.88	29.13	42.93	20.66	48.88	29.04
Gated-Attention	36.06	20.73	48.83	29.10	48.56	20.72	49.21	29.16
**Loss-Attention**	**57.86**	24.88	71.73	**36.95**	**59.38**	25.16	72.52	**37.36**

CapNet								

Dynamic Routing	44.65	22.93	99.91	**37.30**	48.19	22.92	99.99	**37.31**
**Loss-Attention**	**55.60**	23.72	80.11	36.60	**57.65**	23.74	79.12	36.52

**TABLE VI:** Results (%) of Loss-Attention with the Convolutional Architecture on Tiny-Imagenet

Parameter	VGG11_bn					ResNet18				
	Image		Patch			Image		Patch		

	Accuracy	AP	Precision	Recall	F-score	Accuracy	AP	Precision	Recall	F-score

*γ_max_* = 0, *ξ* = 0.1	61.51	55.37	82.27	29.18	43.08	63.68	53.24	82.51	23.09	36.08
*γ_max_* = 0.1, *ξ* = 0	62.15	52.26	82.03	30.91	44.90	63.92	53.26	81.89	31.77	45.78
*γ_max_* = 0.1, *ξ* = 0.1	62.58	57.07	82.67	29.98	44.00	64.05	57.52	82.69	30.71	77.79

**TABLE VII:** Classification Accuracy (%) and Training Time (Seconds) of Loss-Attention With ResNet18 on CIFAR-10 With the Image Size 64 × 64 and Different Stride Values. Note That We Utilize Two GPUs and Set the Batch Size as 48 for a Fair Comparison, “Added” Represents the Added Convolutional Layer, and Time Denotes the Average Time of Model Training for One Epoch. In the First Row, [4] × 1 and [2] × 1 Represent the Added Layer With Stride 4 and 2, Respectively; In the First Column, [2] × *k* (0 ≤ *k* ≤ 3) Means the Number of Layers in the Backbone With Stride 2

Backbone	Added	[4] × 1		[2] × 1	

		Time	Accuracy	Time	Accuracy

[2] × 0		6.58 × 10^2^	92.55	6.60 × 10^2^	92.81
[2] × 1		2.94 × 10^2^	95.27	3.11 × 10^2^	95.19
[2] × 2		1.17 × 10^2^	95.46	1.26 × 10^2^	95.26
[2] × 3		1.04 × 10^2^	95.34	1.06 × 10^2^	95.36

## APPENDIX

### APPENDIX

#### A. Proof of Proposition 1

##### Proof:

Because $p_{m}=h_{m}\theta^{L}$, and Eq. (5) contains $h_{j}\leftarrow \alpha_{j}h_{j}^{L-1}$ and $z=\sum_{m=1}^{M}h_{m}\theta^{L}$, combining these three terms, it has $z=\sum_{m=1}^{M}\alpha_{m}h_{m}\theta^{L}=\sum_{m=1}^{M}\alpha_{m}p_{m}$. Then, a lower bound of the objective in Eq. (2) can be obtained by:

(12)
$$\begin{array}{l} L_{ce}=-log\frac{e^{z_{t}}}{\sum_{k=1}^{K}e^{z_{k}}}\\ \;\;\;\;\;\;\;=-log\frac{e^{\sum_{m=1}^{M}\alpha_{m}p_{mt}}}{\sum_{k=1}^{K}e^{\sum_{m=1}^{M}\alpha_{m}p_{mk}}}\\ \;\;\;\;\;\;\;=-log\frac{\prod_{m=1}^{M}e^{\alpha_{m}p_{mt}}}{\sum_{k=1}^{K}\prod_{m=1}^{M}e^{\alpha_{m}p_{mk}}}\\ \;\;\;\;\;\;\;=-log\frac{\frac{\prod_{m=1}^{M}{\left(e^{p_{mt}}\right)}^{\alpha_{m}}}{\prod_{m=1}^{M}{\left(\sum_{k=1}^{K}e^{p_{mk}}\right)}^{\alpha_{m}}}}{\frac{\sum_{k=1}^{K}\prod_{m=1}^{M}e^{\alpha_{m}p_{mk}}}{\prod_{m=1}^{M}(\sum_{k=1}^{K}e^{{p_{mk})}^{\alpha_{m}}}}}\\ \;\;\;\;\;\;\;=-log\frac{\prod_{m=1}^{M}{\left(q_{mt}\right)}^{\alpha_{m}}}{\sum_{k=1}^{K}\prod_{m=1}^{M}{\left(q_{mk}\right)}^{\alpha_{m}}}\\ \;\;\;\;\;\;\;=-log(1-\frac{\sum_{k=1,k\neq t}^{K}\prod_{m=1}^{M}{\left(\frac{q_{mk}}{q_{mt}}\right)}^{\alpha_{m}}}{1+\sum_{k=1,k\neq t}^{K}\prod_{m=1}^{M}{\left(\frac{q_{mk}}{q_{mt}}\right)}^{\alpha_{m}}})\\ \;\;\;\;\;\;\;\geq \frac{\sum_{k=1,k\neq t}^{K}\prod_{m=1}^{M}{\left(\frac{q_{mk}}{q_{mt}}\right)}^{\alpha_{m}}}{1+\sum_{k=1,k\neq t}^{K}\prod_{m=1}^{M}{\left(\frac{q_{mk}}{q_{mt}}\right)}^{\alpha_{m}}}, \end{array}$$

where the fifth equality is derived from $q_{mt}=\frac{e^{p_{mt}}}{\sum_{k=1}^{K}e^{p_{mk}}}$ and the seventh inequality is on the basis of log(1+a)≤a for all a>−1.

Therefore, Proposition 1 is proved.

#### B. Proof of Proposition 2

##### Proof:

Based on $Z=\sum_{m=1}^{M}\alpha_{m}p_{m}$, a lower bound of the objective in Eq. (3) can be calculate as:

(13)
$$\begin{array}{l} L_{bce}=-\sum_{k=1,y_{k}=1}^{K}log\left(\frac{1}{1+e^{-z_{k}}}\right)\\ \;\;\;\;\;\;\;\;\;\;\;\;\;-\sum_{k=1,y_{k}=0}log\left(1-\frac{1}{1+e^{-z_{k}}}\right)\\ \;\;\;\;\;\;\;\;\;=-\sum_{k=1,y_{k}=1}^{K}log\left(\frac{1}{1+e^{-\sum_{m=1}^{M}\alpha_{m}p_{mk}}}\right)\\ \;\;\;\;\;\;\;\;\;\;\;\;\;-\sum_{k=1,y_{k}=0}log\left(1-\frac{1}{1+e^{-\sum_{m=1}^{M}\alpha_{m}p_{mk}}}\right)\\ \;\;\;\;\;\;\;\;\;=-\sum_{k=1,y_{k}=1}^{K}log\left(1-\frac{e^{-\sum_{m=1}^{M}\alpha_{m}p_{mk}}}{1+e^{-\sum_{m=1}^{M}\alpha_{m}p_{mk}}}\right)\\ \;\;\;\;\;\;\;\;\;\;\;\;\;-\sum_{k=1,y_{k}=0}log\left(1-\frac{1}{1+e^{-\sum_{m=1}^{M}\alpha_{m}p_{mk}}}\right)\\ \;\;\;\;\;\;\;\;\;\geq \sum_{k=1,y_{k}=1}^{K}\frac{e^{-\sum_{m=1}^{M}\alpha_{m}p_{mk}}}{1+e^{-\sum_{m=1}^{M}\alpha_{m}p_{mk}}}\\ \;\;\;\;\;\;\;\;\;\;\;\;\;+\sum_{k=1,y_{k}=0}^{K}\frac{1}{1+e^{-\sum_{m=1}^{M}\alpha_{m}p_{mk}}}\\ \;\;\;\;\;\;\;\;\;=\sum_{k=1,y_{k}=1}^{K}\frac{\prod_{m=1}^{M}{\left(e^{-p_{mk}}\right)}^{\alpha_{m}}}{1+\prod_{m=1}^{M}{\left(e^{-p_{mk}}\right)}^{\alpha_{m}}}\\ \;\;\;\;\;\;\;\;\;\;\;\;\;+\sum_{k=1,y_{k}=0}^{K}\frac{1}{1+\prod_{m=1}^{M}{\left(e^{-p_{mk}}\right)}^{\alpha_{m}}}. \end{array}$$

where the fourth inequality is derived from log(1+a)≤a for all a>−1.

Therefore, Proposition 2 is proved.
